# Consumer Cognition Analysis of Food Additives Based on Internet Public Opinion in China

**DOI:** 10.3390/foods11142070

**Published:** 2022-07-12

**Authors:** Heli Li, Jiyang Luo, Hui Li, Shihe Han, Shuzheng Fang, Li Li, Xuhui Han, Yongning Wu

**Affiliations:** 1 Key Laboratory of Food Quality and Safety for State Market Regulation, Chinese Academy of Inspection and Quarantine, Beijing 100176, China; liheli1234@126.com (H.L.); luojy@caiq.org.cn (J.L.); hanshihe@hotmail.com (S.H.); emorybunton@163.com (S.F.); 2 Beijing Key Laboratory of Diagnostic and Traceability Technologies for Food Poisoning, Beijing Center for Disease Prevention and Control, Beijing 100013, China; lihui@bjcdc.org; 3 Beijing Yunchuang Wangxun Information Technology Co., Ltd., Beijing 100027, China; hanxh@c3in.com; 4 Research Unit of Food Safety, Chinese Academy of Medical Sciences (No. 2019RU014), NHC Key Laboratory of Food Safety Risk Assessment, China National Center for Food Safety Risk Assessment (CFSA), Beijing 100022, China

**Keywords:** network public opinion, food additives, consumer, cognition analysis, risk communication

## Abstract

Food additives play an important role in the food supply, and it has been a food safety topic of great concern to the public. There has been no systematic research on Chinese consumers’ concerns, attitudes, feelings, or opinions on supervision and media coverage of food additives in the past decade, which is an area worth exploring. This study was carried out to deeply understand consumers’ cognition of food additives and formulate food safety risk communication strategies of food additives in China. Big data of consumers’ online public opinion of China on food additives from 2011 to 2020 was collected and cleaned up using Haina Network Public Opinion Monitoring System version 2.0 (HNPOMS V2.0), followed by data analysis and visual display with the Ansi Food Safety Risk Communication System version 2.0 (AFSRCS V2.0). The results showed that the types of food additives of concern to the public have changed from 2011 to 2020, but the amount of food additives has always been of concern. The type of incident that the public is most concerned about is the illegal addition or abuse of additives. The public’s confidence in food production enterprises has been insufficient, but the functions of market supervision are becoming clearer and clearer, and their expectations are constantly increasing. Consumers’ cognition level increases with the strengthening of publicity and popular science, but the influence of “self-media” on public cognition is increasing day by day, and there is cognitive deviation, making it easy to mislead the public. Consumers’ cognition of food additives is the basis of risk communication. Combined with the research results, this paper puts forward corresponding suggestions on the market and social supervision measures, network media guidance strategy and risk communication strategy of China, respectively.

## 1. Introduction

Food additives have gradually developed into one of the indispensable ingredients in modern food processing and play an important role in the food supply. The standard of food additives in production enterprises in China is primarily in accordance with the “GB 2760-2014 National Food Safety Standard—Standard for Uses of Food Additives” [1] and other documents. Although the enforcement of regulatory compliance, such as over range, over limit and illegal addition of additives in food, has been strengthened year by year, food additives are still considered to be unhealthy and even a public health risk by consumers [2]. Studies [3] have shown that the risk cognition of food safety is positively correlated with the risk cognition of food additives. For example, in South Korea, most consumers are concerned about the safety of using food additives in processed foods and do not consider additives to be safe and useful materials in food [4]. Studies [5] have also shown that food additives have become the most concerning factor regarding food safety among Chinese consumers, where 63.6% of surveyed customers claimed to pay attention to food additives before purchasing food products, and 30.7% of surveyed customers demonstrated a lack of objective cognition towards the risks caused by inappropriate use, as well as a low recognition rate of food additives [6,7,8,9]. Consumers believe that artificial flavors may be more acceptable than artificial colors, and factors such as familiarity with ingredients and processing may influence consumers’ decisions on product naturalness [10].

A multi-level analysis of the public’s concerns about food risks in 26 European countries [11] showed that the public’s cognition of food risk is multi-dimensional and complex. Factors such as retail concentration in the food industry and media coverage have contributed to the differences across counties. Consumers’ gender, age, education, income, as well as the level of concern towards food safety, are the factors that significantly affect consumers’ safety risks cognition of food additives [12,13,14]. Females demonstrated [15] a higher level of sensitivity and risk cognition towards food additive safety. In South Korea, it was discovered that different demographic groups share different cognitions towards food additives [4]. For example, nutritional teachers and Non-Governmental Organization (NGO) members seem to be biased against food additives, while food experts are not. According to a report [16], from 2008 to 2013, Korean parents believed that food additives were the most dangerous factor in food, and 82.7% of the surveyed students believed that artificial food coloring was harmful to health [17]. Wu et al. [18] reported that behavioral attitudes, subjective norms, and information cognition had a moderate-to-high impact on food-related panic, and this impact was also transmitted through risk cognition of food additives.

The public’s misunderstanding of food additives may easily lead to public panic or even loss of confidence in food safety overall [19]. Therefore, it has always been a challenge to guide the public to scientifically recognize food additives and reduce cognitive biases [20]. To effectively communicate food safety risk information regarding food additives, the public cognition of food additives must be understood first, and then operative communication strategies can be made. 

Developed countries and regions generally attach importance to the investigation of food safety risk perception in order to understand the public’s risk perception and rules and to guide risk communication activities. For example, since 2005, the European Food Safety Authority (EFSA) has conducted several public food safety risk perception surveys across nearly 30 European countries to systematically capture the public’s risk perception and identify strategic priorities for risk communication [21,22,23]. The EU food safety report [23] was published by the EFSA in 2019, which conducted face-to-face interviews with about 28,000 people from different demographic groups in 28 EU member states using the Standard Eurobarometer surveys. The public’s views on food-related risks and their understanding of the EU’s food safety system were investigated to understand the public’s concerns and regulatory satisfaction with food safety. The U.S. Food and Drug Administration (FDA) has conducted cognitive studies on food-borne diseases and healthy eating behaviors since 1988 [24]. The 2016 food safety survey [25], released by the FDA, reported on consumer food safety risk perceptions, food handling and consumption habits, food counseling, and food-borne illness perceptions. The survey aims to help governments make informed regulations, education and other decisions by better understanding consumer knowledge, attitudes and behaviors related to food safety. The UK Food Standards Agency (FSA) has conducted public food safety surveys to identify the status and trends of public perceptions and attitudes and to assess the process and effectiveness of strategic communication planning since 2001 [26]. In 2009, the German Federal Institute for Risk Assessment (BfR) conducted a special survey [27] on the risk of pesticide residues, which was of great concern to the public. The survey found that the public had a serious lack of basic knowledge of pesticide residues in food, and nearly 70% of the public believed that the use of pesticides in the food was illegal. Based on the results of the survey, BfR improved its communication strategy by focusing on “Laws and regulations on the use of pesticides in food, and the limits of pesticide residues, etc.”, which effectively reduced unnecessary public panic. As early as 1996, some scholars studied the methods of measuring the public’s food safety risk perception from the perspective of psychology and jointly developed the Perceived Food Risk Index questionnaire (PFRI), an in-depth exploration of the model of public perception of food safety risk [28].

In recent years, Chinese scholars have also conducted some research on consumer cognition of food safety, and these studies focus on the impact of food safety risk attributes on consumer cognition, the impact of consumer cognition on consumer trust and consumer behavior, and the causes of cognitive bias. Most of the research is confined to a certain area or a certain risk item [5,6,9,29], which has some reference value for the actual food safety supervision. However, there are still many problems in these studies in China: first, the current studies on the standard of risk perception measurement are insufficient [5]; second, there is an underrepresentation of the population, and nationwide studies on risk perception of food additives in China are rare; third, the depth of the studies needs to be strengthened. Researchers did not explore the internal dimensions of risk perception and proposed targeted communication strategies, which are very limited to the guidance of the national food safety risk communication.

Compared with the United States, Britain, Germany and other developed countries, the food safety risk communication is still in the initial stage in China, and the research is mostly limited to the basic academic stage. There is still a long way to go from the practical application of government supervision, and it is urgent to carry out relevant research effectively in terms of risk communication content, methodology and strategies. Therefore, this study is mainly based on the public information on the Internet of China from 2011 to 2020; uses the Natural Language Processing (NLP) technology and the text mining method to collect and analyze the big data; investigates the cognition of Chinese consumers on food additives and the related factors such as stakeholders in order to provide guidance for the risk communication of food additives; and improves the scientific, targeted and effective of risk communication.

## 2. Materials and Methods

### 2.1. Methods and Tools

This research adopts NLP, text orientation analysis, and word frequency analysis on the basis of the semi-quantitative data research and expert consultation to collect, clean and statistically analyze the big data. The related data processing method is under patent review. Data collection and cleaning were performed via Haina Network Public Opinion Monitoring System version 2.0 (HNPOMS V2.0) [30], Introvision Limited, Beijing, China [31], where a related subject was analyzed based on ‘Food safety knowledge database’, ‘Principal Knowledge database of Weibo’, ‘Ansi Chinese Evaluation Dictionary’, ‘Ansi Chinese recognition dictionary’, ‘Ansi Chinese emotion and attitude dictionary’ using the Ansi Food Safety Risk Communication System version 2.0 (AFSRCS V2.0). The visionary of the information was performed using Excel. 

HNPOMS V2.0 is a self-developed data collection and analysis platform of public opinion by Introvision Limited, Beijing, China. It could locate and follow public opinion related to the object from web information rapidly and accurately, where information sources can be revealed and transmitting subjects can be monitored based on big data and NLP technology. Public opinion can be grasped by multi-dimension visualization and analysis of information to assist scientific decisions. It is characterized by rapid vast data collection, and the preliminary statistical analysis can be performed simultaneously, where the result can be illustrated directly by data modeling, graphics and effect rendering.

AFSRCS V2.0 is a system responsible for the treatment of vast data and information collected by HNPOMS V2.0 and was used to run the multi-dimensional social mentality and public cognition mining analysis of food type and the emotional and attitude cognition dimension based on the databases mentioned above, combined with a deep learning algorithm. The core technology of AFSRCS used a fully intelligent collection method, NLP and intelligent text mining. The related systems and databases mentioned above have applied for software copyright in China.

Introvision Limited, Beijing, has mature research experience in social mentality analysis and public cognitive insight. It has close cooperation with university research institutions and government officials, and most of the research results are confidential. Few public articles [32] can be found on Chinese academic websites.

### 2.2. Data Sources

The data in this study were mainly retrieved from three main media platforms, Weibo (2011–2020), Toutiao (2017–2020) and Douyin (2018–2020), covering the period from 1 January 2011 to 30 June 2020. A total of 5255 hot topics and 265,799 comments related to food additives were retrieved. Correlation analysis [33,34] of retrieved topics and comments was conducted using the keywords “food additives”, “manufacturers”, “regulators”, “media”, “popular science publicity”, “emotion” and “attitude”.

## 3. Results and Analysis

### 3.1. Consumers’ Cognition of Food Additives

#### 3.1.1. Types of Food Additives 

**Figure 1:**
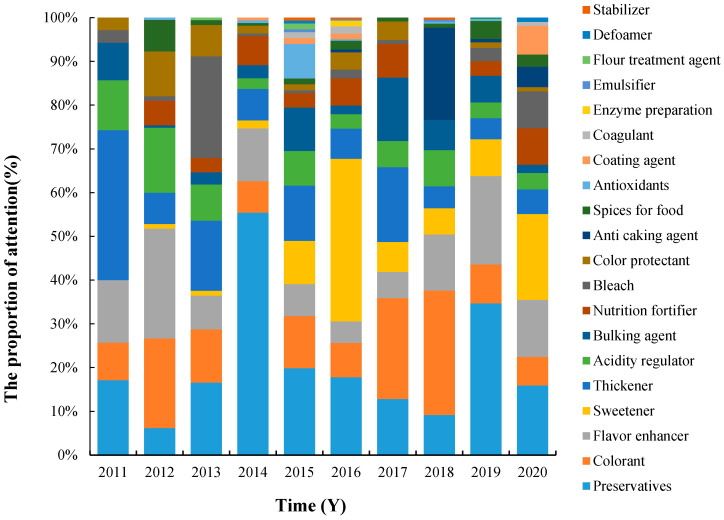
Consumers’ attention to various food additives from 2011 to 2020.

This study shows that from 2011 to 2020, the types of additives of concern to consumers changed from year to year. With the year 2018 as the dividing point, the overall category of concern has been characterized from centralized to decentralized, which may be related to national policy regulation or news hot spots. During this period, several common food additives such as preservatives, colorants, flavor enhancers, sweeteners, and thickeners have been receiving significant attention, as shown in Figure 1. Among them, more attention was paid to “preservatives” in 2014, mainly because the topics such as “China Food and Drug Administration (CFDA) notified that the preservatives of Holilai moon cakes exceeded the standard” [35] caused widespread public attention. At that time, CFDA conducted annual risk monitoring on food and drugs, notifying consumers of any risk items found in food. In 2016, consumers’ attention focused on “sweeteners”, mainly related to the mass dissemination of topics such as “Citrus cyclamate? Are farmers’ revelations credible?” [36] In 2018, “colorants” attracted more attention, mainly due to related incidents such as “Tens of thousands of tons of dried persimmons in Guangxi were illegally dyed” [37]. In 2019, the hot topic “A piece of American pastry has not gone bad for more than 40 years” [38] made “preservatives” once again the focus of the public. 

At the same time, something interesting can be observed from this graph, which is that if an additive receives a lot of attention in one year, then its attention drops off rapidly for at least another year after that. Similar to the preservatives mentioned above, it received a lot of attention in 2014, but in 2015–2018, the attention declined rapidly. This should be the result of the joint efforts of the government and the production enterprises.

Studies have shown that food additives receiving the most attention in South Korea are preservatives, synthetic flavorings, colorants [16], artificial sweeteners [39], and artificial food colorants [17].

#### 3.1.2. The Dosage of Food Additives 

From 2011 to 2020, the dosage of additives has always been a topic of concern and hot discussion among consumers, with related comments comprising more than 70% of all comments. A relatively consistent and stable cognition has been established that “dosage is the main factor leading to frequent issues of food additives”. Many consumers have expressed that “toxicity must be considered in the context of dosage, so treat it rationally”, “everything is toxic, and the dosage makes all the difference” [40], etc. This is consistent with the research results of Bearth et al. [41]. By allowing consumers to understand topics such as maximum allowed dosage of additives and scientific evaluation, consumers expressed more positive thoughts and feelings, with less risk cognition and higher acceptance. However, many consumers still have questions such as “how the reasonable or harmless dosage of additives was determined” [42]. In addition, there are certain differences in consumers’ cognition of the “scope of use”. Some consumers expressed that additives within a certain standard range are acceptable, while some others believed that additives should be absolutely prohibited and not be used under any scope.

### 3.2. Consumers’ Cognition of Food Production

#### 3.2.1. Illegal or Abusive Use of Additives in Food Production

In early 2011, the “Emergency Notice on Strengthening the Supervision and Administration of Food Seasonings and Food Additives” [43] jointly issued by relevant authorities of China set clear requirements for food safety supervision. Since then, the media and consumers have also been actively uncovering related issues on food additives, promoting widespread public concern.

The data show that the number of topics related to food additives fluctuated around 600 from 2013 to 2020, with high popularity. The related topics, ranking from high to low, are: (1) the use of additives in food production, sales, catering services, etc.; (2) popular science publicity from media or experts; (3) the introduction of relevant policies and regulations; (4) the results that research institutions released. Among them, the most popular topics regarding the use of additives is in food production related to the illegal addition of non-edible substances to food, abuse of food additives, and other incidents. These may be explained by two reasons: first, the early regulatory compliance, including policies and standards in China towards food additives, were not comprehensive enough; second, after the major food safety incident of “Sanlu Milk Powder” in 2008, the rectification of violations related to the addition of non-edible substances to food and food additive abuse became a focus of regulatory authorities.

#### 3.2.2. Consumers’ Lack of Confidence in Food Production Enterprises

From 2011 to 2020, as the directly responsible party for the production/operation/use of additives, the trends of related enterprises have always attracted the attention of consumers. The enterprises mentioned are mostly condemned and criticized by consumers for abusing food additives and illegally adding non-edible substances. The proportion of mentions of market supervision departments shows a steady increase, especially in 2018 and 2019. The institutional reform of market supervision departments in 2018 resulted in high consumer expectations and increased attention, which is consistent with the results described in Section 3.3.1.

**Figure 2:**
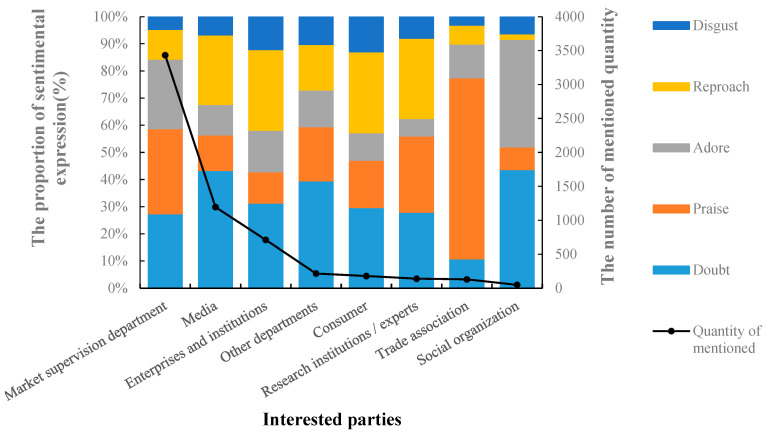
Main emotion of consumers toward the interested parties.

The main emotional expression of consumers to the interested parties is shown in Figure 2. Consumers’ emotional expression toward the market supervision departments and the media ranked first and second, respectively, among which the positive emotions, such as praise and adore, toward the market supervision departments accounted for more than 50%. Negative emotions such as doubts and reproaches about the media were high. Consumers’ praise and adoration for the industry association is particularly prominent, which is the first positive emotion obtained by all interested parties. Judging from 2019 to 2020, consumers still have strong negative sentiments towards enterprises and institutions, and especially for food production enterprises, consumers are full of doubts and distrust. Some comments include expressing that with so many morally corrupted merchants in the industry, it is difficult to guarantee food safety. Thus, it is hard for some consumers to change their negative impressions of food companies, and it is a long way to go to regain consumers’ confidence.

### 3.3. Government Regulation

#### 3.3.1. The Market Supervision Function

**Figure 3:**
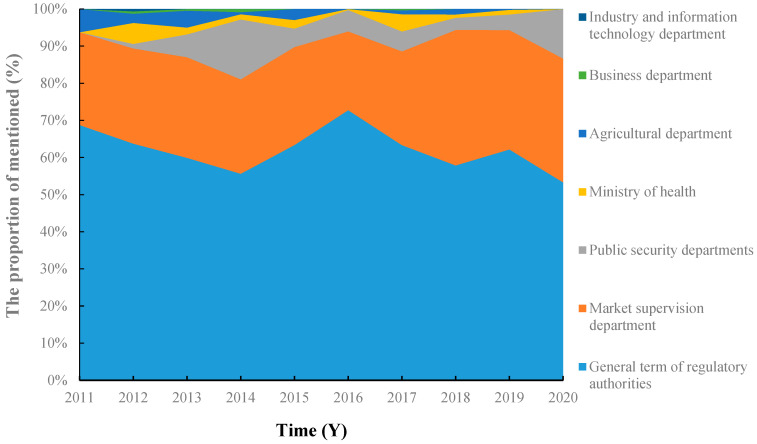
Trends in consumer perceptions of the regulatory authorities from 2011 to 2020.

Consumers often refer to the regulatory authorities as “government”, “relevant departments”, and “administrative departments”, as shown in Figure 3, which indicates that most consumers have only a vague recognition of food additive regulatory authorities.

From 2011 to 2020, consumers’ attention to market supervision departments has gradually increased, especially in 2018 and 2019. A similar trend is observed for the media, mainly focusing on topics related to the 3.15 Gala. Among them, the “Walnut Peanuts” [44] exposed in 2018, the “Natural Eggs” [45] and “Shrimp and Egg Spicy Strips” [46] exposed in 2019 caused the public’s dissatisfaction with the market supervision departments. Some of the comments expressed that “The responsible department highlighted in the 3.15 Gala should be held accountable” and so on. The influence of the 3.15 Gala not only brought CCTV and other authoritative mainstream media to the forefront, but the work of market supervision departments has also received more and more attention. Consumers have a clearer understanding of the responsibilities of the departments. At the same time, their expectations for market regulation continue to rise, with more stringent requirements.

#### 3.3.2. Consumer Cognition Improves with the Strengthening of Publicity and Popularization

According to the data, topics related to popular science publicity have increased significantly, which started gradually attracting consumers in 2014. In 2018, the proportion of topics around safety evaluation and standards gradually increased.

**Figure 4:**
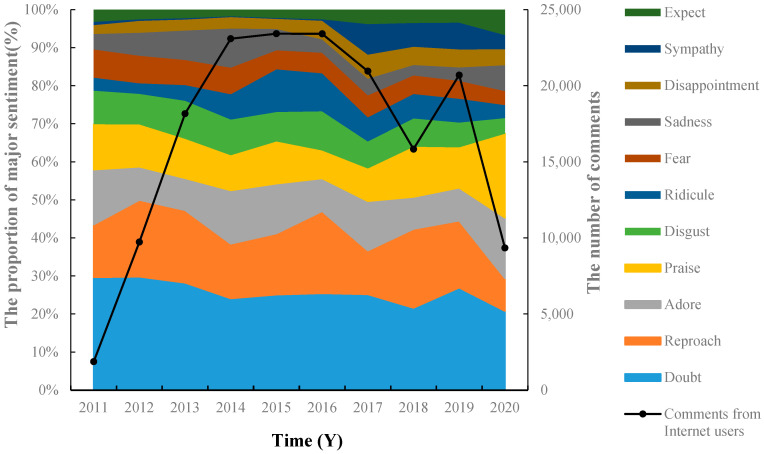
Trend of consumer’s emotion about additive issues from 2011 to 2020.

From 2011 to 2020, in the discussion of food additive-related topics, the proportion of “doubt” always ranks first among all categories of sentiments, as shown in Figure 4. Many consumers can accurately express their doubts, such as “Why add preservatives to food” [47] and so on.

It is worth noting that the proportion of negative sentiments, including “doubt”, “reproach”, and “disgust”, has gradually decreased. The proportion of “praise” sentiment has shown a trend of increase since 2017. This was manifested as: firstly, satisfaction and recognition of popular science publicity and media exposure; and secondly, support and approval for relevant policies, regulations and regulatory measures. In December 2019, the Chinese State Administration for Market Regulation issued an announcement on “Strengthening the quality and safety supervision of seasoned flour products” [48], which gained widespread support from consumers. This indirectly shows that the public’s satisfaction with the food additives is improving, the popularization of science has achieved remarkable results, and consumers’ cognition of food additives and their understanding and support of relevant policies and regulations have increased. Previous studies [2] have shown that the improvement of consumers’ knowledge weakens the risk cognition of food additives. In food safety incidents, knowledge has a buffering effect on the negative impact of food additives [49]. South Korea’s 2008–2013 food additive safety assessment report [16] showed that both parents and children have a high demand for food additive education and publicity information and are interested in food additive safety, legal standards, and food containing food additives. A study [39] has evaluated the impact of information dissemination (such as leaflets and pamphlets) on consumers’ knowledge, behavior, and safety cognitions of preservatives. The results showed that the knowledge score before and after information dissemination increased from 67.3% to 91.9%. Safety cognition scores increased significantly, showing a pre- and post-test difference of 60%.

In this study, some consumers expressed “adore” with respect to food additives and related comments such as “Food additives are not bad, to ensure texture and quality “ [50] and so on. On the one hand, it reflects, to a certain extent, that with the deepening of the public’s understanding of food additives, the phenomenon of “turning pale at the mention of additives” in the society has decreased. On the other hand, it also shows that after reading a large number of reports, some consumers have gradually become indifferent and have paid little attention to the possible harm of food additives.

### 3.4. The Influence of Media and Self-Media

#### 3.4.1. Self-Media Has an Obvious Influence on Public Cognition

From 2011 to 2020, the number of popular micro-blogs related to food additives published by self-media and central media was relatively large. Among them, the number of related popular micro-blogs published by the self-media accounted for 40.7%. The central media accounted for 23.4%. Online business media and local media accounted for 13.1% and 12.80%, respectively.

According to the statistics of the top ten micro-blogs published by various media related to food additives, the number of related popular Micro-blogs from self-media showed an obvious trend of increasing from 2011 to 2020. From 2011 to 2017, the central and local media received more attention, and the number of relevant popular Micro-blogs peaked in 2014, mainly regarding the reports or alerts of food safety incidents related to food additives. From 2017 to 2019, the number of popular micro-blogs for food additives increased significantly. Self-media and online business media have gradually developed into the main sources of opinions. There was a sharp decrease in the number of popular micro-blogs from central and local traditional mainstream media, compared to previous years.

Reports on food additives from self-media are more recognized by the public than the ones from traditional media. Judging from the attitude of consumers, the sentimental expression of “praise” and “adore” toward the self-media is much higher than that of traditional media, while the expression of “reproach” is lower than that of the traditional media. It can be summarized that some consumers firstly believed that some media lacked scientific literacy and the reports on food additives were seriously misleading. Relevant comments include “Reporters’ scientific literacy needs to be improved” [51], “Products that meet quality certification and additives that are legally used should not be demonized” [52]. Secondly, consumers suspected that some media are deliberately exaggerating the toxicity of food additives or maliciously smearing them. The authority and credibility of the mainstream media have been questioned and challenged.

#### 3.4.2. The Media’s Abilities of Publicity and Interpretation Need to Be Improved, and Some Self-Media Has a Biased Cognition

**Figure 5:**
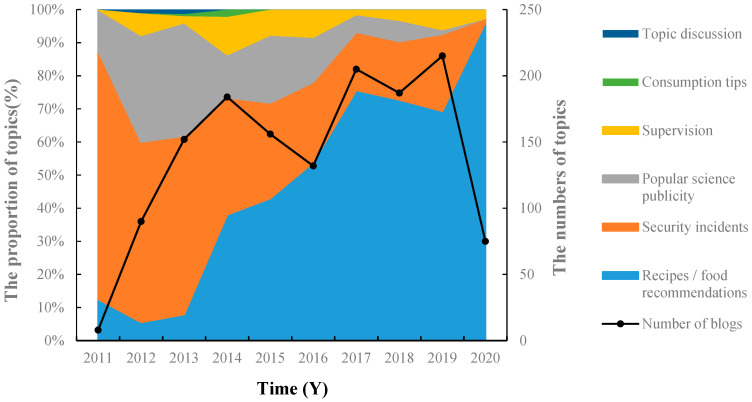
Trends in consumer concerns about food additive-related topics from 2011 to 2020.

From 2011 to 2020, it was shown from the consumers’ attention to topics related to food additives in the media that consumers pay insufficient attention to related topics that guide and enhance the public’s awareness of food additives, such as “supervision and management”, “consumption tips”, and “popular science publicity”. The relevant micro-blogs accounted for a small proportion of the hot spots in recent years, showing a downward trend year after year, as shown in Figure 5. The topics on media regarding supervision and popular science topics received less and less attention. The media’s role in bridging needed to be improved. The possible reasons are: firstly, the decrease in interest in food additive incidents in recent years has led to fewer related topics; secondly, consumers are less interested in traditional food additive popularization content; thirdly, lack of in-depth reporting and interpretation from media perspectives as the media have mainly retransmitted the voice of the regulatory authorities in recent years.

Popular media articles on healthy food production have a potential impact on public cognition of food additives. Recipe/food recommendation topics account for about 50% of the healthy food production category, and such topics have increased rapidly since 2013 (Figure 5). It is worth noting that such articles often emphasize the absence of additives. For example, “Mother teaches me how to pickle radishes like this. It is really affordable and hygienic. There are no additives, and it is a must for the winter” [53]. This had an unintentional magnifying effect on the public’s risk cognition of food additives. Many self-media reports have an unclear understanding of the principles of use of food additives, and their publications are seriously misleading. A large number of claims from self-media, such as “no additives”, “no coloring”, “no preservatives” or “not any additives are used” invisibly deepens the public’s misunderstanding that “food containing food additives is unsafe”.

## 4. Discussion and Conclusions

Studies investigating food safety risk perception have substantially increased in recent years, particularly because of recent cross-disciplinary development. In terms of research methods, this study used different methods from those reported in the past, such as the Standard Eurobarometer surveys [23], the PFRI [28], and structured or semi-structured interviews [54], etc. Similar to the report of Nardiv et al. [55], this study used mathematical statistics based on big data.

In the Netherlands, food experts indicated [54] that low reliability in the food industry and the preponderance of negative information about food additives on the internet and social networks are the main causes of high-risk perception. It was reported [56] that the difficulty in pronouncing the name of food additives was related to the perception of their risk in the United States. The harder it is to pronounce, the more the substance is perceived as harmful to health. However, this study was challenged in 2017 [57], suggesting that risk perception was more related to the size of the word than to the difficulty of pronouncing it. 

There are countless similar studies, which can explain that the factors that affect consumers’ perception of food safety risk may vary according to different survey times, different places or different survey methods, the causes of which may be intrinsically linked with people’s living habits, thinking patterns, living environment and others.

A survey in South Korea [58] showed that in 2013, television was considered the most appropriate channel to communicate the risk of food additives, which is consistent with the survey results in China in 2014 [59]. In 2011, the authors of [39] showed that nearly half of the respondents chose leaflets and pamphlets as the preferred media for information dissemination. The news media plays a critical role in building the public’s cognition of food safety. In 2015, a study [60] showed that media reports have a social amplification effect. Media exposure is negatively correlated and one of the key factors in consumers’ attention to food-related risks. Chen et al. [7] reported that the public’s high susceptibility to the risk of food additives is a result of trust in food safety-related work from the government and enterprises instead of a lack of conceptual knowledge.

In conclusion, the results of this study suggest that Chinese consumers’ perceptions of food additives are influenced by stakeholders such as food manufacturers, regulators and the media. From a scientific point of view, the cognition of Chinese internet consumers on food additives and their influencing factors in the last ten years were obtained. It is hoped that the results can provide some data support for regulators, guide the direction of future risk communication, and better serve consumers.

### 4.1. Limitations

A limitation of the study is that the object of this study is only limited to Chinese Internet users. The research results can only represent the cognitive situation of the general population in China, such as their views, emotions and attitudes, without a detailed analysis of other factors such as gender, age, occupation, income and other demographic characteristics. The research is the collection and analysis of the accumulated data on the network, and it is macro and general. More professional and academic research needs to be carried out through future investigations.

### 4.2. Suggestions and Further Research

Drawing on existing researches [18,61,62], it is recommended that relevant state departments establish an effective food safety risk communication mechanism; release food safety supervision information in a timely and accurate manner; strengthen the prevention and control of food production enterprises for “illegally adding and abusing food additives”; strengthen the awareness of the responsibility of additives users; promote self-discipline of industry associations and enterprises; encourage the media and the public to supervise and expose illegal activities, and combine multiple departments and channels to achieve social co-governance.

Raising public awareness of food additives is key for the Chinese government to formulate and implement food safety risk management policies. To meet the increasingly higher expectations from the society, it is recommended that relevant state departments improve strategies for communicating with the public and strengthen the guidance to the public on rational cognition of food additives [29,63]. Some recommendations include firstly that relevant departments produce easy-to-understand short videos or animated promotional videos so that consumers can obtain correct knowledge through authoritative channels; meanwhile, for hot topics related to food additives, respond to public concerns to gain trust in their abilities; punish violations with a firm attitude to resonate with consumers and gain trust and show concern. Secondly, conduct a targeted information exchange for different users of network platforms, such as Micro-blog, WeChat, Zhihu, and Bilibili, in which consumers’ concerns, tastes, and contextual expressions are very different. Targeted publicity and interaction should be carried out in different environments according to differences in consumer groups and food additive topics. Targeted communication strategies should be developed toward food additive-related risks [4], improving the effects of risk communication while covering more consumers. Thirdly, encourage industry enterprises to actively participate in popular science publicity, set good examples of reputable enterprises, publicize their scientific and standardized use of food additives through vivid stories and live cases, respond to the public’s doubts about critical food safety issues, change consumers’ stereotypical pursuit of “zero food additives”, and improve the public’s cognition towards food additives and trust in enterprises.

At the same time, it is recommended to attach importance to the guidance, supervision and management of self-media and online media and curb misleading reports of public food safety risk information by the media.

In addition, it is also suggested that the Chinese government officially carry out purposeful investigations and research on food safety risk perception by institutions such as the EFSA, the BfR and the FSA, timely publish the investigation results, and conduct risk communication with the international community.

The results of this study are of reference significance in the formulation of China’s national food safety risk communication policies, and it is more purposeful and directional to guide the future food safety risk communication in China. In the next research plan, we expect to carry out food additive risk communication activities with different themes for different groups of people, such as activities to introduce food safety knowledge to campuses, provide risk interpretation of food additives to residents in the community, introduce typical cases of illegal addition or abuse of food additives in enterprises, and regularly communicate with the media about food additives and popularize knowledge, etc., to investigate people’s risk perception of food additives before and after risk communication, so as to measure the effect of food safety risk communication, and timely adjust the corresponding policies.

## Data Availability

The data presented in this study are available on request from the corresponding author.
